# Internal Transcribed Spacer and 16S Amplicon Sequencing Identifies Microbial Species Associated with Asbestos in New Zealand

**DOI:** 10.3390/genes14030729

**Published:** 2023-03-16

**Authors:** Erin Doyle, Dan Blanchon, Sarah Wells, Peter de Lange, Pete Lockhart, Nick Waipara, Michael Manefield, Shannon Wallis, Terri-Ann Berry

**Affiliations:** 1 Applied Molecular Solutions Research Centre, Te Pūkenga–New Zealand Institute of Skills and Technology, Private Bag 92025, Auckland 1142, New Zealand; edoyle@unitec.ac.nz (E.D.); svanwinkel@unitec.ac.nz (S.W.); pdelange@unitec.ac.nz (P.d.L.); 2 School of Environmental and Animal Sciences, Te Pūkenga–New Zealand Institute of Skills and Technology, Private Bag 92025, Auckland 1142, New Zealand; 3 Institute of Fundamental Sciences, College of Sciences, Massey University, Palmerston North 4442, New Zealand; p.j.lockhart@massey.ac.nz; 4 The New Zealand Institute for Plant & Food Research Limited, Mt Albert, Auckland 1142, New Zealand; nick.waipara@plantandfood.co.nz; 5 Water Research Centre, School of Civil and Environmental Engineering, University of New South Wales, Sydney, NSW 2052, Australia; manefield@unsw.edu.au; 6 Environmental Solutions Research Centre, Te Pūkenga–New Zealand Institute of Skills and Technology, Private Bag 92025, Auckland 1142, New Zealand; swallis@unitec.ac.nz (S.W.); tberry@unitec.ac.nz (T.-A.B.)

**Keywords:** asbestos, bioremediation, chrysotile, fungi, bacteria, siderophores, amplicon sequencing, New Zealand

## Abstract

Inhalation of asbestos fibres can cause lung inflammation and the later development of asbestosis, lung cancer, and mesothelioma, and the use of asbestos is banned in many countries. In most countries, large amounts of asbestos exists within building stock, buried in landfills, and in contaminated soil. Mechanical, thermal, and chemical treatment options do exist, but these are expensive, and they are not effective for contaminated soil, where only small numbers of asbestos fibres may be present in a large volume of soil. Research has been underway for the last 20 years into the potential use of microbial action to remove iron and other metal cations from the surface of asbestos fibres to reduce their toxicity. To access sufficient iron for metabolism, many bacteria and fungi produce organic acids, or iron-chelating siderophores, and in a growing number of experiments these have been found to degrade asbestos fibres in vitro. This paper uses the internal transcribed spacer (ITS) and 16S amplicon sequencing to investigate the fungal and bacterial diversity found on naturally-occurring asbestos minerals, asbestos-containing building materials, and asbestos-contaminated soils with a view to later selectively culturing promising species, screening them for siderophore production, and testing them with asbestos fibres in vitro. After filtering, 895 ITS and 1265 16S amplicon sequencing variants (ASVs) were detected across the 38 samples, corresponding to a range of fungal, bacteria, cyanobacterial, and lichenized fungal species. Samples from Auckland (North Island, New Zealand) asbestos cement, Auckland asbestos-contaminated soils, and raw asbestos rocks from Kahurangi National Park (South Island, New Zealand) were comprised of very different microbial communities. Five of the fungal species detected in this study are known to produce siderophores.

## 1. Introduction

Asbestos is the commercial name for a group of six naturally-occurring, fibrous silicate minerals in the amphibole (actinolite, amosite, anthophyllite, crocidolite, and tremolite) or serpentine (chrysotile) groups [1,2]. Historically, chrysotile, crocidolite, and amosite have been the most widely used worldwide because of their strength, heat, chemical resistance, and electrical insulation properties [3,4,5,6].

Inhalation of asbestos fibres can cause lung inflammation and later development of asbestosis, lung cancer, and mesothelioma [7]. This has been linked experimentally in part to iron at the surface of asbestos fibres generating hydroxyl radicals and reactive oxygen species (ROS), which cause DNA damage [2,7,8]. For this reason, the use of asbestos is banned in many countries (approx. 35%, [9]) but continues to be used in others, particularly countries that still mine substantial quantities of asbestos (e.g., Russia and China) [10]. In most countries, large amounts of asbestos exists within building stock, buried in landfills, and in contaminated soil [10,11]. Mechanical, thermal, and chemical treatment options do exist, but these are prohibitively expensive, and they are not effective for contaminated soil, where only small numbers of asbestos fibres may be present in a large volume of soil [2,12].

Research has been underway for the last 20 years into the potential use of microbial action to remove iron and other metal cations from the surface of asbestos fibres to reduce their toxicity [11]. All six types of asbestos contain iron, either as part of the fibre structure, or in the case of chrysotile, replacing Mg^2+^ or Si in the outer layer of the fibre [13,14]. Iron is an essential nutrient for bacteria, fungi, and plants, but it is often not readily biologically available due to its relatively low solubility [2,15,16]. To access sufficient iron for metabolism, many microbes produce organic acids, or iron-chelating siderophores [2,3].

Lichens are well-known for their production of biologically-active secondary metabolites and for their ability to weather rocks [17]. Favero-Longo et al. (2005) [11] noted that lichens often colonised asbestos cement roofs and asbestos-containing rocks, and studied the effects of four lichen species (*Candelariella vitellina*, *Lecanora rupicola*, *Xanthoparmelia pulla* and *Xanthoparmelia tinctina*) on substrates containing asbestos. They observed that fungal hyphae penetrated more than 2 mm into the rock, surrounding individual fibres. They also found that magnesium was selectively removed from chrysotile fibres, and attributed this to the production of oxalic acid by the different lichen species. Experiments with solutions of oxalic acid and chrysotile fibres showed reduced free radical generation, most likely due to the removal of iron and magnesium, and the disruption of the silicate structure of the chrysotile fibres [11]. Further in vitro studies using the isolated mycobiont from *X. tinctina* with chrysotile fibres found that the fungus surrounded the fibres with hyphae, released oxalic acid, depleted magnesium from the fibres, and produced oxalates at the interface between the fungus and chrysotile [18]. The confirmation that an isolated lichen fungus can modify chrysotile in vitro is important because lichens are communities made up of a fungus (mycobiont) and one or more photobionts (green algae and/or cyanobacteria) as well as potentially surface basidiomycete yeasts [19], filamentous non-lichenised fungi [20], and bacteria [21], any of which may have an effect on the degradation of asbestos fibres.

An issue with the use of lichens for bioremediation is their slow growth rate and incomplete coverage of asbestos rock surfaces [11]. Free-living fungi generally have a faster growth rate than lichenized fungi, and a small number of fungal species have been found to produce siderophores and/or degrade asbestos fibres in vitro. For example, Martino et al. (2003) [22] found that the soil fungi *Geomyces pannorum*, *Mortierella hyalina*, *Oidiodendron maius*, *O. griseum*, and *Fusarium oxysporum* were able to extract iron from crocidolite in culture through the secretion of siderophores. Similarly, Daghino et al. (2005) [23] found that *F. oxysporum* successfully removed iron from fibres of crocidolite, amosite, and chrysotile, inhibiting free radical release. Borges et al. (2022) [24] showed that *Aspergillus niger* was able to degrade chrysotile fibres in vitro.

To increase the possibility of finding siderophore-producing fungi, researchers progressed from using general soil organisms to ones found in asbestos mine tailings. Daghino et al. [23,25] found that isolates of *Verticillium* and *Paecilomyces* from asbestos mine soils were effective at removing iron from crocidolite and chrysotile. Despite the harsh environmental conditions in asbestos deposits, fungal diversity can be relatively high. Daghino et al. (2008) [26] isolated a large number of saprotrophic fungi from two abandoned asbestos mines in northern Italy, finding 60 different isolates at one site, and 38 at the other, including species of *Aspergillus*, *Cladosporium*, *Epicoccum*, *Mortierella*, *Myrothecium*, *Paecilomyces*, *Penicillium*, and *Verticillium*. Twelve of these were screened for the ability to produce siderophores, and eleven were found to do so. Three of these species (*Aspergillus fumigatus*, *Paecilomyces lilacinus* and *Verticillium leptobactrum* were able to remove iron from chrysotile in vitro. Daghino et al. (2009) [27] found that *V. leptobactrum* was a dominant species at one asbestos mine and three other serpentinite sites, and was able to solublise Si, Fe and Mg from chrysotile fibres. In India, Bhattacharya et al. (2016) [28] found six fungal isolates from rocks and soil from four abandoned asbestos mines. Two were identified as *Aspergillus tubingensis* and *Coemansia reversa*, and both were able to produce siderophores in vitro.

Bacteria are also known to produce organic and other acids to release mineral nutrients and siderophores to extract iron and other metal ions [2,16]. Borges et al., 2022 [24] reported that the soil bacterium *Acidothiobacillus thiooxidans* is able to degrade asbestos and asbestos cement through the production of sulphuric acid. *Pseudomonas* species are known to produce a range of siderophores, can decrease the iron content in asbestos cement, and the isolated bacterial siderophores pyoverdine and pyochelin are effective at removing iron from chrysotile, crocidolite, and amosite fibres [2]. Bacterial isolates from rocks and soil in asbestos mines are able to reduce the iron content of asbestos fibres in vitro [3]. Bacterial growth rates are generally faster than fungi [3], but fungi are considered to remove metals from silicates faster than bacteria [29], and fungal siderophores may be more effective than bacterial ones [15].

To ensure the success of microbial bioremediation in a field situation, it is essential to start with a large group of candidate siderophore-producing fungi or bacteria that are a good match for the environmental conditions of the area. A sensible approach is to survey sites containing asbestos, such as mine sites, weathered building materials, and contaminated soils [10]. Being able to culture bacteria or fungi is an essential step for further trials, but for fungi at least only a small fraction of species can be readily cultured, potentially excluding useful species [30]. DNA-based methods such as high-throughput amplicon sequencing can reveal such “hidden’ diversity and can facilitate the culturing of fungi with very specific environmental requirements once we know they are present [31].

This paper uses internal transcribed spacer and 16S amplicon sequencing to investigate the fungal and bacterial diversity found on naturally-occurring asbestos minerals, asbestos containing building materials, and asbestos-contaminated soils.

## 2. Materials and Methods

### 2.1. Sampling Procedure

**Figure 1:**
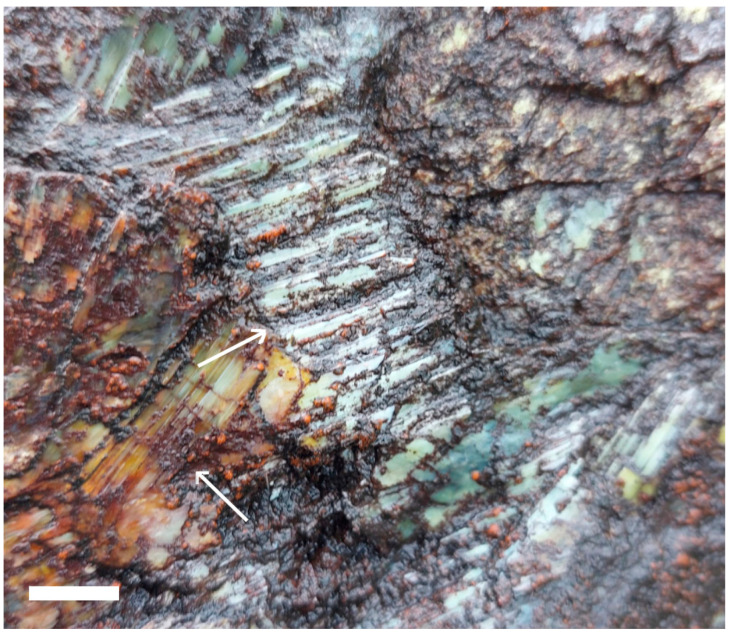
Biofilm on asbestos material, Kahurangi National Park, Nelson, New Zealand. Arrows show areas of biofilm development on asbestos (chrysotile) fibres. Scale bar = 5 mm.

**Figure 2:**
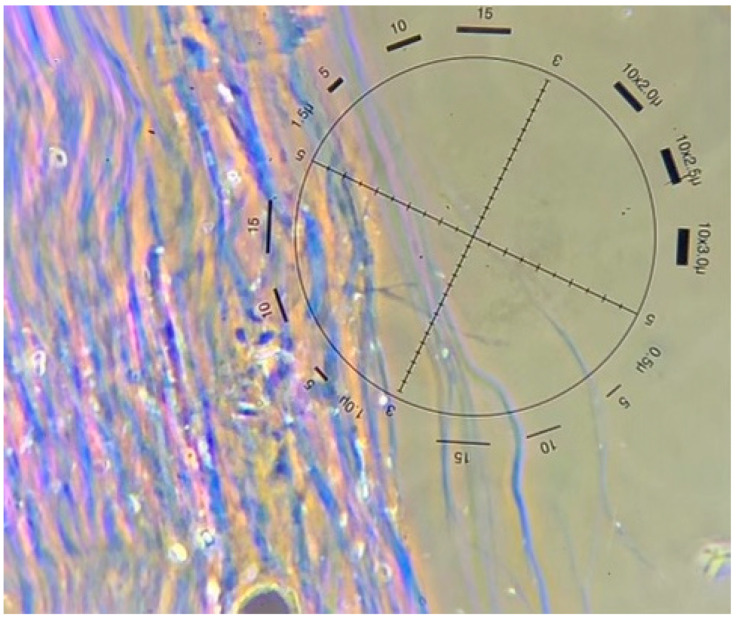
Chrysotile fibres from a soil sample seen with polarized light microscopy and dispersion staining (image courtesy of Focus Analytics, 2023).

Samples of biofilms were taken from asbestos mine tailings, asbestos-contaminated soil, and asbestos-containing building materials (ACM), such as cladding and Super 6 roofing. Ten spatially separated biofilm samples were taken during a site visit to an asbestos mine in Kahurangi National Park, South Island, New Zealand (−41.123107° S 172.699993° E, 611 m a.s.l.) in February 2022. Rocks lying within the mine tailings and exposure face containing asbestos fibres were identified visually, and most of these were found to have a black, brown, or orange biofilm growing on them (Figure 1). Sterile cotton swabs were used to sample the biofilms. These were stored in plastic bags at 4 °C for 36 h, and then frozen at −80 °C until DNA extraction could be carried out. Biofilm samples from asbestos cement building materials and asbestos-contaminated soils were taken from pre-identified sites in Auckland, New Zealand, by licensed removalists whose clients had agreed to the use of their samples for further testing. Soil samples were collected from private properties throughout the Auckland region by 4SIGHT Consulting following established health and safety protocols for asbestos sample collection. Bulk soil samples were collected from three sites in the Auckland region, with 1–4 samples per site. One 1.5 mL subsample was taken from each of these bulk soil samples and stored at –20 °C for later DNA extraction. Bulk samples were tested for the presence of asbestos by Focus Analytics using low powered stereomicroscopy followed by polarised light microscopy including dispersion staining techniques (AS 4964-2004) (Figure 2).

### 2.2. DNA Extraction

DNA extractions were carried out inside a negative pressure unit, following safety protocols for the handling of asbestos-containing materials [32]. DNA was isolated from biofilm swabs collected from the ACM and the asbestos mine tailings using the DNeasy^®^ Plant Mini Kit (Qiagen, Germantown, Maryland, United States of America). The cotton tip was cut from the wooden handle of the swab and incubated in AP1 Buffer for three hours at 65 °C before being removed from the sample tubes. The extraction was then carried out as per the manufacturer’s recommendations, with the DNA being eluted into 50 µL of AE buffer. DNA was extracted from the soil samples using the NucleoSpin^®^ Soil Kit (Macherey-Nagel, Düren, Germany) following manufacturer’s recommendations, with a final elution volume of 100 µL. After the extractions were completed, the DNA sample tubes were wiped down with a wet wipe before being removed from the NPU.

### 2.3. Sequencing

To identify the diversity of fungi present within each sample, a 500–600 bp section of the internal transcribed spacer (ITS) region was amplified via PCR using the primers ITS1F, 5′-CTT GGT CAT TTA GAG GAA GTA A and ITS2 5′-GCT GCG TTC TTC ATC GAT GC (Integrated DNA Technologies, Coralville, IA, USA) [33]. PCRs were performed in 25 µL reactions containing 13 µL of ultra-pure water, 0.5 µL of each primer, 10 µL of Platinum™ Green Hot Start DNA Polymerase (Thermofisher Scientific, Suzhou, China), and 1 µL DNA template. DNA was denatured for 95 °C for 2 min, followed by 40 cycles of 95 °C for 30 s, 48 °C for 30 s, and 72 °C for 1 min, followed by a final extension period of 10 min at 72 °C. In addition, a 290–295 bp section of the bacterial 16S ribosomal RNA region was amplified using the primers 515F, 5′-GTG YCA GCM GCC GCG GTA A and 806R, 5′-GG ACT ACN VGG GTW TCT AAT [34]. PCRs contained 13 µL of ultra-pure water, 0.5 µL of each primer, 10 µL of Platinum™ Green Hot Start DNA Polymerase (Thermofisher Scientific, Suzhou, China), and 1 µL DNA template. Samples were denatured at 94 °C for 3 min, followed by 35 cycles of denaturation at 94 °C for 45 s, annealing at 50 °C for 60 s and extension at 72 °C for 90 s, and then a final extension period at 72 °C for 10 min. PCR products were run on a 2% agarose gel and visualized using an Uvidoc HD6 (Uvitec Ltd., Cambridge, United Kingdom) to confirm amplification success. The ITS and 16S PCR amplicons from each sample were pooled, and then purified using AMPure XP magnetic beads and eluted into ultra-pure water prior to Illumina MiSeq™ sequencing at Massey Genome Service, Massey University, Palmerston North, New Zealand.

### 2.4. Data Processing

Illumina MiSeq 300 bp paired-end reads were processed for quality using a standard Illumina sequence analysis pipeline. Reads were trimmed to their longest contiguous segment for which quality scores were less than a quality cutoff, set at 0.01, using the dynamictrim application from the SolexaQA++ software (http://solexaqa.sourceforge.net/, accessed 8 September 2022) (Figures S1 and S2). Further processing was performed in R v4.2.2. ITS and 16S reads were demultiplexed using the functions filterFastq in the package ShortRead [35] and fastqPairedFilter in dada2 [36]. Sequences were then further processed following the dada2 amplicon sequencing workflow. Primer sequences were removed using Cutadapt v.4.1, paired-end reads were merged using mergePairs, and chimeric sequences were removed using the removeBimeraDenovo function. We computed read-based rarefaction curves using the function rarecurve in the R package *vegan* [37] for each sample to examine whether all ASVs in the sample had been sequenced. Sequencing effort can be considered sufficient to detect all ASVs when the curve reaches an asymptote. Taxonomic identifications of the 60 most abundant amplicon sequence variants (ASVs), which were present in three or more samples, from both the 16S and ITS datasets were confirmed using the National Center for Biotechnology Information (NCBI) BLASTn algorithm. A relatively conservative approach was employed when assigning names to ASVs from NCBI BLAST search results. Identification at the species level was made if there was a match of 99% or above to a reference sequence for that species, with no other close matches. Where two or more species were a 100% match, the sequence was assigned an identification at the genus level. Where there were no close matches to one species but multiple matches (>95%) to species in different genera, identification was made to the family level. Raw reads of the biofilm sample ITS and 16S rRNA gene amplicons have been deposited in the NCBI Sequence Read Archive (SRA) and are available under the Project accession number PRJNA940522.

To test the hypothesis that fungal and bacterial communities differ between the three sample types, the Bray–Curtis dissimilarity index was calculated for log(10)+1 transformed ASVs abundance data from the ITS and 16S datasets. Differences in community composition were then examined by ordination and visualised with non-metric multidimensional scaling (MDS) using the function *metaMDS* in the R package *vegan* [37]. The goodness-of-fit of each ordination was assessed by examining stress levels.

## 3. Results

### Characterisation of Amplicon Sequence Variants (ASVs)

Species accumulation curves show asymptotes for all samples in both the ITS and 16S datasets regardless of sample type (Figure S3), indicating that the data provide a good description of the diversity present in the samples, with all ASVs sequenced. These rarefaction curves also show the high variation in diversity between samples. Although this variation was not overtly related to sample type, ACM samples tended to have low ITS diversity but high 16S diversity compared to other samples.

After filtering, the ITS dataset consisted of 490,663 reads belonging to 819 ASVs across the 36 samples (Table 1). ITS read counts for individual samples varied from 958 to 30,159 reads, with a mean of 13,629.53 (SD 7678.44). Six of these ASVs, identified as *Cladosporium cladosporioides, Epicoccum nigrum*, *F. oxysporum*, *Alternaria alternata*, *Pseudopithomyces chartarum*, and *Vishniacozyma* sp. were present in all three sample types. No ITS ASV was present in all samples. After processing and filtering, one asbestos-contaminated soil sample (Soil02) did not contain any 16S reads despite successful amplification of the PCR product, leaving 35 samples in the dataset. There were 287,686 reads in the 16S dataset, belonging to 1003 ASVs (Table 2). 16S read counts for individual samples ranged from 1632 to 14,454 reads, with a mean of 8219.6 (SD 3068.42). One 16S ASV, which could not be identified, was common to all three sample types. The samples taken from ACM contained more unique 16S ASVs than the other sample types, with 608 of the 639 (95.15%) ASVs found among these samples not being found in the other sample types. Conversely, the ACM samples in the ITS dataset had the lowest proportion of unique ASVs, with 324 of 354 (66.1%) of the ASVs not being found in the other sample types. The samples taken from raw asbestos contained more unique ITS ASVs than the other sample types, with 269 of the 287 (93.73%) ASVs not being found in the other sample types.

**Figure 3:**
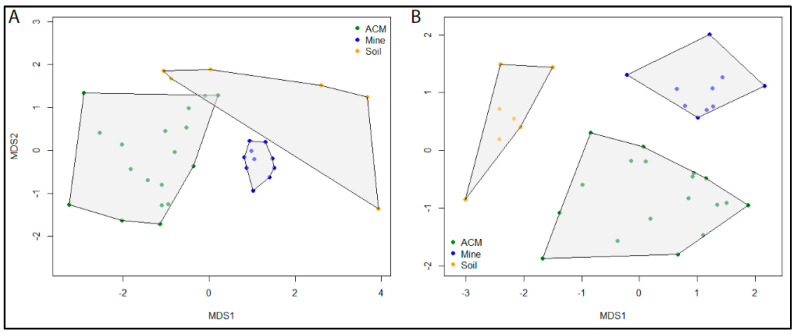
Ordination plots using non-metric multidimensional scaling (MDS) using Bray–Curtis dissimilarity from log(10)+1 transformed abundances from (**A**) ITS ASVs from 35 samples and (**B**) 16S ASVs from 36 samples identified by metabarcoding of ITS and 16S datasets, respectively. Samples are colour coded according to sample type: ACM (green), asbestos contaminated material; Mine tailings (blue), raw asbestos samples from mine tailings; Soil (orange), asbestos contaminated soil samples.

The initial MDS analysis of the ITS data resulted in strong clustering of all samples, with the exception of a single outlier. The samples Soil03 and Soil09 both contained a single ASV. However, the ASV in Soil09 was present in eight other samples, preventing it from becoming an outlier in the analysis. To improve the clarity of the plot, the Soil03 outlier was removed from the analysis. MDS plots showed distinct separation of communities according to sample type for both the fungal and bacterial communities. Stress levels were 0.14 for both the ITS and 16S datasets, indicating a good agreement of the data to the model predictions. Samples from asbestos mine tailings showed the greatest similarity among samples of the same type and also the greatest dissimilarity to samples of other types (Figure 3). Bacterial communities from asbestos-contaminated soil samples were also highly unique (Figure 3B). In contrast, fungal communities from asbestos-contaminated materials and asbestos-contaminated soil samples (Figure 3A) as well as bacterial communities from asbestos-contaminated materials (Figure 3B) were all highly variable. However, these samples still displayed a greater degree of similarity to samples of the same type than to other sample types.

**Figure 4:**
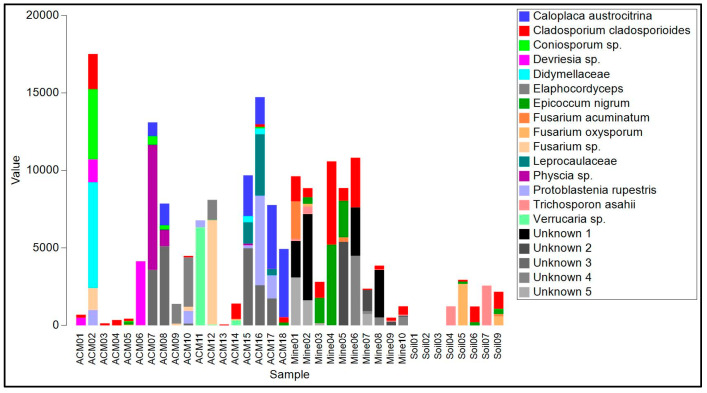
Read frequency of the 20 most abundant amplicon sequence variants (ASVs) from the ITS region found in each sample.

The biofilms on ACM consisted of lichenized fungi (*Protoblastenia rupestris*, species of *Physcia* and *Verrucaria*, and an unknown species from the Leprocaulaceae), filamentous ascomycete fungi (mainly species of *Cladosporium*, *Coniosporum*, *Devriesia*, *Elaphocordyceps*, and *Neodevriesia*), an ascomycete yeast (*Salinomyces thailandica*), a basidiomycete yeast (*Vishniacozyma carnescens*), and a filamentous basidiomycete (*Wallemia muriae*) (Table S1, Figure 4). The bacterial component of these biofilm samples (Table S2, Figure 4) were mainly actinomycetes, particularly from the genus *Rubrobacter*, with occasional unidentified members of the Deinococcales and a proteobacterial species in the genus *Sphingomonas*. Cyanobacteria, when present, were species of *Chroococcidiopsis* or *Macrochaete*.

**Figure 5:**
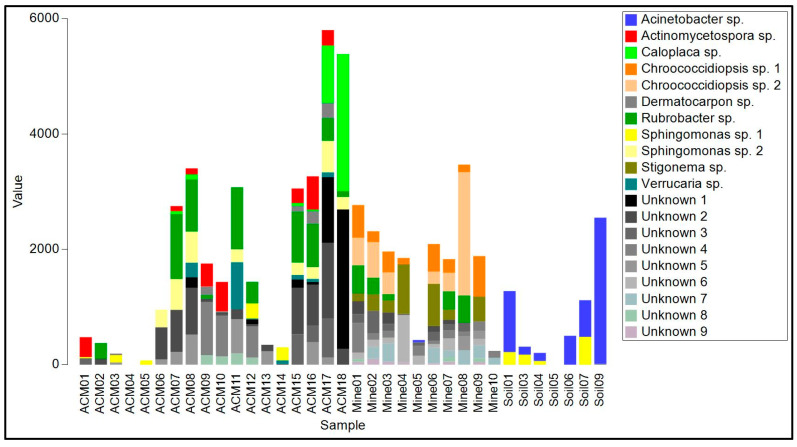
Read frequency of the 20 most abundant amplicon sequence variants (ASVs) from the 16S region found in each sample.

The raw asbestos fibre biofilm samples showed little evidence of colonization by lichenized fungi (one species of *Lecidea* was detected in three samples), but filamentous ascomycete fungi were common (mainly *A. alternata*, *C. cladosporioides*, *E. nigrum* and *Fusarium acuminatum*), as were basidiomycete yeasts (*Sporobolomyces ruberrimus*, *Trichosporon asahii*, and *Yarrowia lipolytica*) and basidiomycete polypore fungi (*Fomitopsis hemitephra* and *Ganoderma australe*) (Table S1, Figure 4). The bacterial component of the biofilm (Table S2, Figure 5) was dominated by cyanobacteria, mainly *Chroococcidiopsis*, but also *Stigonema* and an unknown species in the Nostocales. Non-photosynthetic bacteria included species of *Spirosoma* and the actinomycete *Rubrobacter*.

Asbestos-contaminated soil samples were found to contain a mixture of filamentous ascomycete fungi (*C, cladosporioides*, *C. sphaerosporum*, *E. nigrum*, *F. acuminatum*, *F. oxysporum* and a species of *Neodevriesia*), basidiomycete yeasts (*Saitozyma podzolica*, *T. asahii*, *Vishniacozyma carnescens*, and *Y. lipolytica*), and a filamentous basidiomycete (*W. muriae*) (Figure 4, Table S1). No lichenized fungi were detected. Bacteria present included *Actinobacter johnsonii*, *Acidovorax facilis*, species of *Masilia*, *Rheinheimera*, and the actinomycete *Arthrobacter* (Figure 5, Table S2). No cyanobacteria were detected.

## 4. Discussion

The MDS analyses indicate that the microbial communities from Auckland ACM and the soil samples and Kahurangi National Park asbestos rocks are significantly different, with little overlap in community structure between sample types. However, community composition within each sample type was also highly variable, particularly for fungal and bacterial communities originating from asbestos contaminated materials, and fungal communities from asbestos contaminated soils. In contrast, samples from the asbestos mine tailings were relatively similar, particularly for the fungal communities. The finding that the biofilm swabs from Auckland asbestos cement, Auckland asbestos-contaminated soils, and raw asbestos rocks from Kahurangi National Park were comprised of very different microbial communities is unsurprising, as Auckland soils, asbestos cement building materials, and asbestos/serpentinite rocks have dissimilar chemical properties (particularly pH). In addition, the geology, vegetation cover, and environmental conditions in Kahurangi National Park are very different to those in Auckland. There, the asbestos occurs as chrysotile manifest as cross fibre and/or slip fibre stockworks, or, less commonly, agglomerates of finely-matted chrysotile fibre in the associated serpentinised dunite rock [38,39] surrounded by native forest, whereas in Auckland the asbestos is found in an urban context.

Most of the fungal species found on ACM are xerophilic and/or halotolerant and have previously been recorded from stonework. For example, the lichen *P. rupestris* has been recorded as spreading on concrete surfaces in the Auckland region [40], and the basidiomycete yeast *S. thailandica* is frequently isolated from stone monuments and is known to be halotolerant [41]. Most of the bacterial genera present in the ACM biofilm samples have previously been isolated from soil samples in New Zealand [42]. Cyanobacteria were not commonly detected, which may be due to unfavourable pH, moisture, and temperature conditions. Interestingly, one bacterium, *Actinomycetospora iriomotensis*, has previously been isolated from a lichen sample [43], and it may have come from one of the lichens growing on the ACM.

Serpentinite and asbestos deposits are harsh environments for plants and microbial species, in part because they are nutrient-poor, have major cation imbalances, high pH (10–12), and high concentrations of phytotoxic trace elements, notably nickel [44]. Several of the fungi detected in the biofilms from the asbestos mine in Kahurangi National Park have previously been recorded from serpentinite, including *Cladosporium cladosporioides*, *F. oxysporum* [26], and *S. ruberrimus* [45]. The presence of two basidiomycete polypore species (*F. hemitephra* and *G. australe*) may suggest that spores or other material from those species in the surrounding forest are being deposited in the biofilms. The abundance of cyanobacteria in the biofilm samples at this site is likely due to Kahurangi National Park being a high light, high rainfall area.

Most of the fungal species found in the asbestos-contaminated soil samples have been reported from soil previously in the literature. For example, *Cladosporium cladosporioides* is a decomposer of organic material in soils with a worldwide distribution [46], *E. nigrum* is routinely isolated from plant material and air and soil [47], *F. oxysporum* is found throughout the world (as a soil saprophyte or plant endophyte or plant disease, depending on isolate) [48], and *Sa. podzolica* is a widespread soil yeast [49]. It was surprising that bacterial diversity was fairly low in the asbestos-contaminated soil samples. This may be due to different conditions at each of the sampling sites, but could also be due to the lower total read count caused by filtering of lower quality sequences for these samples, which may have provided insufficient information for ASV identification.

The generation of a dataset of microbial species found on asbestos-containing substrates now allows the use of more selective isolation and culturing methods to target species with very specific environmental requirements [30,31]. Five of the fungal species detected in this study (*Cladosporium cladosporioides*, *E. nigrum, F. oxysporum*, *S. ruberrimus*, and *Y. lipolytica*) have been already been reported in the literature as being able to produce siderophores in vitro [26,42,50,51]. Once a large range of microbial species are successfully grown in culture, production of siderophores will be detected by culturing candidate microbial species on chrome azurol S (CAS)-agar plates [52]. Fungi and/or bacteria that are capable of producing siderophores can then be tested with asbestos fibres in in vitro experiments to test their ability to reduce their toxicity.

## 5. Conclusions

For microbial bioremediation to be successful in a field setting, it is crucial to begin with a large pool of potential siderophore-producing bacteria or fungi that are well suited to the environmental conditions of the area. Our investigation of locations that contain asbestos, such as mining sites, weathered building materials, and contaminated soils has revealed a diverse range of fungal and bacterial species, five of which have previously been reported as producing siderophores in the literature. The use of high-throughput amplicon sequencing to identify microbial species associated with asbestos now allows us to revisit the original sampling sites and use isolation and culturing methods that match the specific environmental requirements of potentially useful microbial species. Once in culture, these species will be screened for siderophore production, and tested with asbestos fibres in vitro.

## Figures and Tables

**Table 1:** Summary of ASV and read numbers, post-processing, for each sample type within the ITS dataset.

	ACM (n = 18)	Raw (n = 10)	Soil (n = 8)
**Unique ASVs**	324	269	192
**Total ASVs**	353	287	221
**Total Reads**	241,028	175,706	73,929
**Mean ASVs per sample**	27.67 (SD = 14.79)	42.7 (SD = 13.58)	31.38 (SD = 42.68)
**Mean reads per sample**	13,390.44 (SD = 6943.3)	17,570.6 (SD = 6309.29)	9241.13 (SD = 8263.27)

**Table 2:** Summary of ASV and read numbers, post-processing, for each sample type within the 16S dataset.

	ACM (n = 18)	RAW (n = 10)	Soil (n = 7)
**Unique ASVs**	608	225	136
**Total ASVs**	639	245	154
**Total Reads**	169,465	83,691	36,880
**Mean ASVs per sample**	50.94 (SD = 18.85)	45.7 (SD = 11.55)	28.29 (SD = 14.35)
**Mean reads per sample**	9414.72 (SD = 2793.59)	8368.2 (SD = 2387.1)	4932.86 (SD = 2956.69)

## Data Availability

Raw reads of the biofilm sample ITS and 16S rRNA gene amplicons have been deposited in the NCBI Sequence Read Archive (SRA) and are available under the Project accession number PRJNA940522.

## Supplementary Materials

The following supporting information can be downloaded at: https://www.mdpi.com/article/10.3390/genes14030729/s1, Table S1: Taxonomic identifications of the 60 most abundant amplicon sequence variants (ASVs) for ITS and read numbers for each biofilm sample; Table S2: Taxonomic identifications of the 60 most abundant amplicon sequence variants (ASVs) for 16S and read numbers for each biofilm sample. Figures S1 and S2: Frequency histograms of forward and reverse read lengths produced from paired-end Illumina MiSeq amplicon sequencing of ITS and 16S; Figure S3: Rarefied species accumulation curves by read count in the (A) ITS dataset, and (B) 16S dataset.
