# Supplementary material for: Internal Transcribed Spacer and 16S Amplicon Sequencing Identifies Microbial Species Associated with Asbestos in New Zealand
Source: Genes (Basel). 2023 Mar 16;14(3):729. doi: 10.3390/genes14030729 (PMC10048439; doi:10.3390/genes14030729)
Supplement: Supplementary file 1 [file genes-14-00729-s001.zip › genes-2236387-supplementary.pdf]

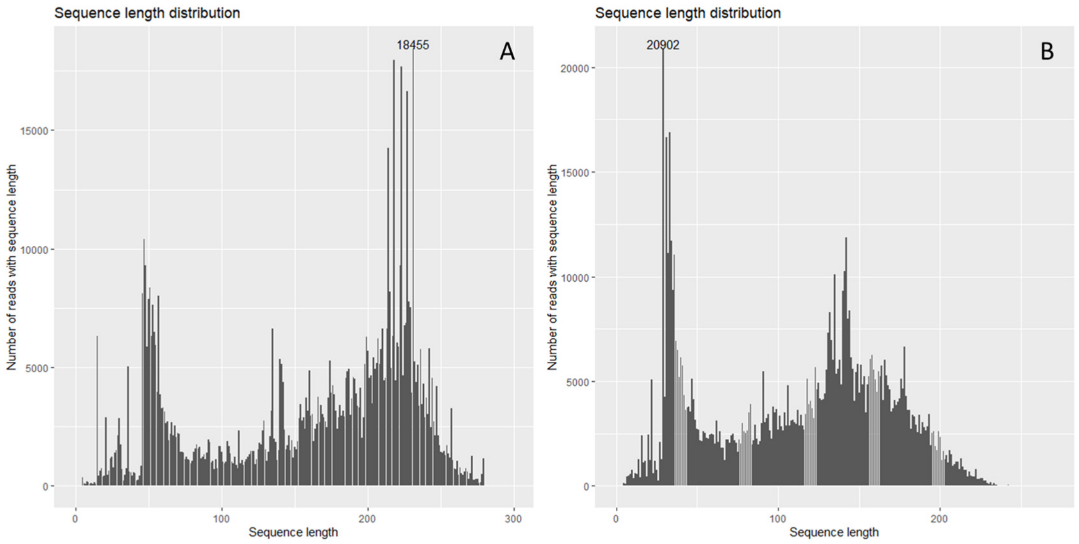

Figure S1: Frequency histogram of forward (A) and reverse (B) read lengths produced from paired-end Illumina MiSeq amplicon sequencing of ITS. Note, reads lengths are subsequent to primer removal and initial trimming for quality.

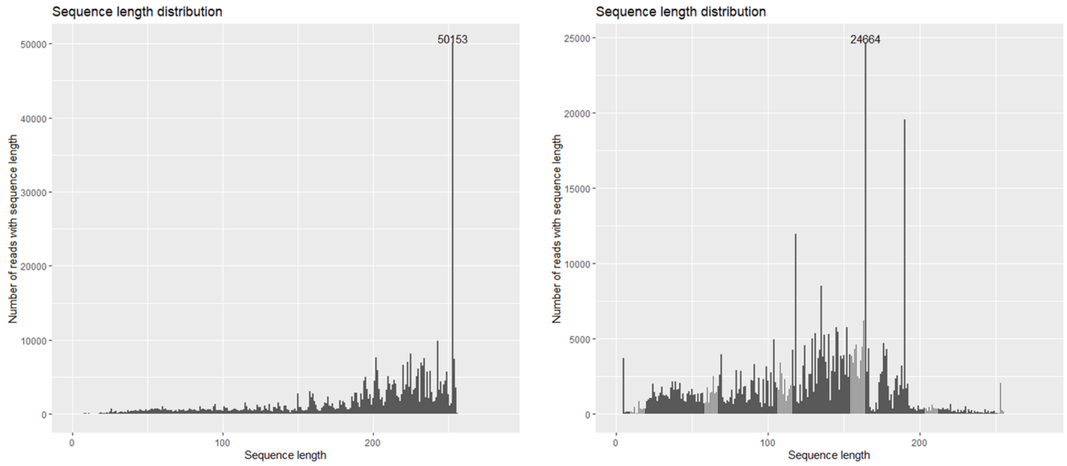

Figure S2: Frequency histogram of forward (A) and reverse (B) read lengths produced from paired-end Illumina MiSeq amplicon sequencing of 16S. Note, reads lengths are subsequent to primer removal and initial trimming for quality.

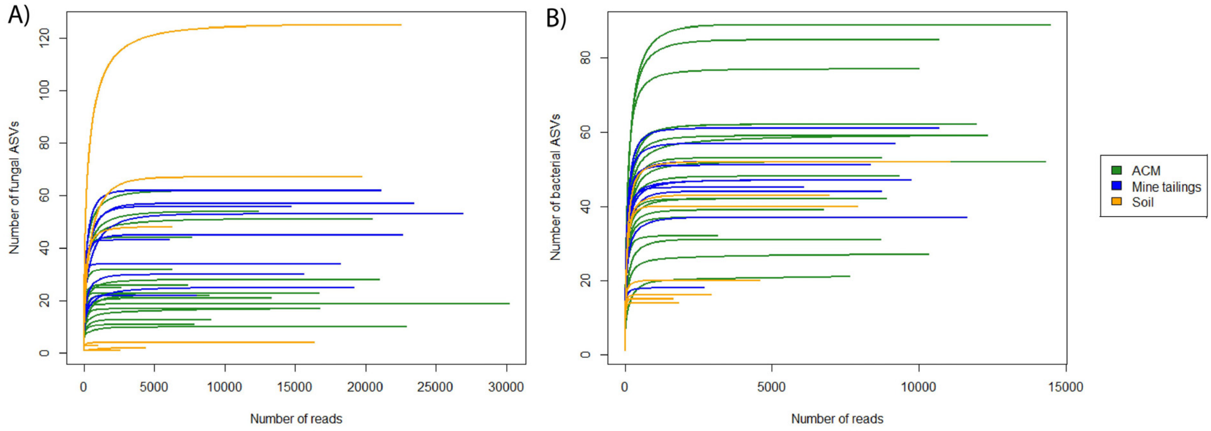

Figure S3: Rarefied species accumulation curves by read count in the A) ITS dataset, and B) 16S dataset. ACM (green), asbestos contaminated material; Mine tailings (blue), raw asbestos samples from mine tailings; Soil (orange), asbestos contaminated soil samples.

Table S1: Abundances of the most abndance 60 ITS ASVs, with identifications determined based on high percentage sequence matches with reference sequences on NCBI.

**ITS ASVs**	**ACM**	**Mine**	**Soil**
Alternaria alternata	61	2591	110
Biscogniauxia sp.	0	632	0
Bullera alba	0	489	0
Caloplaca austrocitrina	15208	0	0
Capnodiales sp.	648	0	0
Cladosporium cladosporioides	4753	13687	2232
Cladosporium sp.	0	501	0
Cladosporium sphaerospermum	1558	0	677
Coniosporum sp.	5465	0	33
Cyphellophora sp.	2280	0	0
Devriesia sp.	6104	0	0
Didymellaceae	7588	0	0
Elaphocordyceps sp.	5770	0	0
Epicoccum nigrum 1	455	9618	656
Epicoccum nigrum 2	0	8227	0
Fomitopsis hemitephra	0	3505	0
Fungi 1	0	14116	0
Fungi 2	0	1501	0
Fungi 3	0	1429	0
Fungi 4	935	0	0
Fungi 5	0	7111	0
Fungi 6	18144	0	0
Fungi 7	0	5744	0
Fungi 8	0	5561	0
Fungi 9	0	2340	0
Fungi 10	4614	0	0

Fungi 11	0	2937	0
Fungi 12	2141	0	0
Fusarium acuminatum	0	2718	154
Fusarium gramineum	543	0	0
Fusarium oxysporum	120	183	3262
Fusarium sp.	8528	0	0
Ganoderma australe	0	698	0
Helotiales sp.	2087	0	306
Hymenochaetales sp. 1	0	600	0
Hymenochaetales sp. 2	0	403	0
Lecidea sp.	0	2810	0
Leprocaulaceae	5738	0	0
Meyerozyma guilliermondii	0	558	0
Neodevriesia sp 2	1311	0	132
Neodevriesia sp. 1	1875	0	0
Neodevriesia sp. 3	1336	0	0
Paraphoma sp.	415	0	0
Physcia sp.	9233	0	0
Plectosphaerella sp.	157	0	454
Preussia minima	112	397	0
Protoblastenia rupestris	9702	0	0
Pseudopithomyces chartarum	901	808	451
Salinomyces thailandica	725	0	0
Sporobolomyces ruberrimus	0	3120	0
Stereaceae	0	1465	0
Trichosporon asahii	0	783	3808
Verrucaria sp. 1	6743	0	0
Verrucaria sp. 2	1709	0	0
Verrucariaceae	1342	0	0
Vishniacozyma carnescens	145	0	1424
Vishniacozyma sp.	12	332	66
Wallemia muriae	399	0	510
Yarrowia lipolytica 1	0	1391	2314
Yarrowia lipolytica 2	0	806	365

Table S2: Abaundaces of the most abndance 60 16S ASVs, with identifications determined based on high percentage sequence matches with reference sequences on NCBI.

**16S ASVs**	**ACM**	**Mine**	**Soil**
Acinetobacter sp. 1	0	38	5008
Acinetobacter sp. 2	0	127	556
Acinetobacter sp. 3	0	56	1074
Actinomycetospora sp.	2537	0	0
Archaea	1550	0	0
Bacteria 1	5759	607	0
Bacteria 2	1618	1174	0

Bacteria 3	2151	1478	0
Bacteria 4	2215	371	0
Bacteria 5	0	1703	0
Bacteria 6	0	1505	0
Bacteria 7	632	200	0
Bacteria 8	0	3887	0
Bacteria 9	0	1501	0
Bacteria 10	0	498	0
Bacteria 11	0	316	0
Bacteria 12	0	1172	0
Bacteria 13	0	1077	0
Bacteria 14	0	805	0
Bacteria 15	168	591	0
Bacteria 16	657	0	0
Bacteria 17	913	0	0
Bacteria 18	0	747	0
Bacteria 19	0	509	0
Bacteria 20	96	77	0
Bacteria 21	229	0	0
Bacteria 22	0	1630	0
Bacteria 23	1609	0	0
Bacteria 24	0	890	0
Bacteria 25	0	1301	0
Bacteria 26	788	0	0
Bacteria 27	905	0	0
Bacteria 28	0	939	0
Bradymyces sp.	341	110	0
Caloplaca sp.	3588	0	0
Chroococcidiopsis sp. 1	0	2781	0
Chroococcidiopsis sp. 2	0	4136	0
Chroococcidiopsis sp. 3	0	3449	0
Chroococcidiopsis sp. 4	0	2020	0
Chroococcidiopsis sp. 5	0	1513	0
Chroococcidiopsis sp. 6	748	0	0
Cyanobacterium	4026	0	0
Dermatocarpon sp.	769	0	14
Dothideomycetes sp.	694	0	0
Fibrella sp.	1128	0	0
Fungi	0	352	0
Hymenobacter sp.	635	0	0
Prokaryote	1220	64	169
Pseudonocardia sp.	914	0	0
Ralstonia sp.	114	0	596
Rubrobacter sp.	5956	1660	0
Sphingomonas sp. 1	705	0	942
Sphingomonas sp. 2	705	0	0
Sphingomonas sp. 3	2784	0	0

Spirosoma sp. 1	0	1315	0
Spirosoma sp. 2	0	775	0
Stigonema sp.	0	2839	0
Verrucaria sp. 1	1346	0	0
Verrucaria sp. 2	3574	0	0
Verrucaria sp. 3	414	0	0
